# Immigrants to the United States contribute to society: Here are 3 ways to support their transition

**DOI:** 10.1093/haschl/qxae019

**Published:** 2024-02-14

**Authors:** Rita Hamad

**Affiliations:** Department of Social and Behavioral Sciences, Harvard School of Public Health, Boston, MA 02115, United States

**Keywords:** immigration, policy, health disparities

## Abstract

The number of migrants entering the United States in 2023 shattered records. Despite prevailing narratives, immigrants, on average, contribute substantially to US society. Rather than slamming the door in the faces of newcomers, federal, state, and local policymakers should provide services to these individuals to ensure they have the maximum opportunity to thrive, both for their own benefit and for the greater social good. Public health and social science research provides ample rigorous evidence on the benefits of different types of investments in these vulnerable individuals upon their arrival in our country. In this Commentary, I review 3 examples of potential evidence-based investments: social inclusion, meeting basic needs, and supportive neighborhoods.

## Introduction

The number of migrants crossing the southern US border in recent months has shattered records, with over 177 000 arrested by Border Patrol in August 2023, and at least 91 000 arriving as part of a family group.^1^ This does not include thousands more arriving through other routes. Many are asylum seekers fleeing war or violence, and others do not meet this official definition but are hoping to escape climate crises or economic deprivation and seek new hope in the fabled Land of Opportunity. Despite prevailing narratives, immigrants, on average, contribute substantially to US society.^2^ One exemplary study documented that immigrants put in $115 billion more to the Medicare Trust Fund than they took out in 2002–2009, in contrast to the deficit generated by US-born people.^3^

Rather than slamming the door in the faces of newcomers, federal, state, and local policymakers should provide services to these individuals to ensure they have the maximum opportunity to thrive, both for their own benefit and for the greater social good. Immigration law itself is a well-documented social determinant of health,^4^ and public health and social science research provides ample rigorous evidence on the benefits of other varied types of investments in these vulnerable individuals upon their arrival in our country. Below I review 3 examples of potential evidence-based investments, ranging from social inclusion and meeting basic needs to ensuring supportive neighborhoods.

On a personal note, I am an immigrant to this country. My family was part of the wave of refugees from the Middle East that I now study in some of my own research. Fleeing civil war, we lived in several Middle Eastern countries—including in the Persian Gulf during the 1991 Gulf War—before moving permanently to the United States in the 1990s. My parents received the Earned Income Tax Credit and food stamps (now known as the Supplemental Nutrition Assistance Program [SNAP]). While we have also faced our share of government-sanctioned hardships, the support that my family received in our first years in the United States helped us to thrive, and ultimately enabled me to now conduct research on the same safety-net policies I once received, from a place of privilege in academia.

## Social inclusion

Research in the social sciences suggests that social inclusion of refugees and other vulnerable immigrants has pay-offs for economic and social outcomes as well. These benefits accrue to both the immigrants themselves as well as their broader communities. For example, one study exploited a natural experiment created by a California policy allowing undocumented immigrants to obtain driver's licenses, finding that it improved traffic safety, specifically by reducing the number of hit-and-run accidents.^5^ Another study in several major US cities found that supporting naturalization of eligible immigrants not only increased their earnings and homeownership rates but also increased tax revenues in their cities of residence and, in most cases, decreased the overall cost of public benefits.^6^ This report conducted by the Urban Institute was sponsored by the Office of Immigrant Affairs in New York City, which is among the cities most affected by the current influx of asylum seekers, and where political debates are raging about how far to go in accommodating these newcomers.

While there is more limited quasi-experimental research on how US policies that support social inclusion of at-risk immigrants influence health, a handful of studies have suggested the potential benefits. For example, studies of the Deferred Action for Childhood Arrivals (DACA) program—a 2012 policy that prevented deportation for undocumented immigrants who came to the United States as children—have demonstrated that it resulted in improved mental health for both the DACA beneficiaries themselves during adulthood, as well as their own children.^7,8^

## Social safety net benefits

One major area where governmental support of refugees and other immigrants is lacking in the United States is access to the social safety net. With a few exceptions, the United States does not allow most immigrants—even those with legal documentation—to benefit from government policies to support low-income families.^9^ Only citizens and “resident aliens” (including permanent residents and others with “substantial presence”) are eligible for the Earned Income Tax Credit, the largest US poverty alleviation program.^10^ SNAP provides nutrition assistance to “lawfully present non-citizens” who meet certain complex criteria, often including a lengthy work history. Similarly, Temporary Assistance for Needy Families (TANF; a short-term income support program with substantial work requirements) as well as Medicaid and the Children's Health Insurance Program are only available to citizens or “qualified” non-citizens; even lawful permanent residents have a 5-year waiting period before gaining access to these benefits.^11^ The Child Tax Credit is available to parents of any immigration status, but their children must have a social security number.^12^ One of the few programs with more flexible immigration criteria is the Special Supplemental Nutrition Program for Women, Infants, and Children (WIC), which is available regardless of immigration status and has been shown to improve birth outcomes for children of low-income immigrant women.^13^ Yet, WIC only provides nutrition support to low-income pregnant and postpartum women and children under 5 years. Moreover, WIC take-up rates for older children are woefully low, with only 25% of eligible 4-year-olds enrolled in the program.^14^ For several of these programs that are administered by states, some states provide the benefits to immigrants who are ineligible based on federal guidelines as long as they meet income and other eligibility requirements.^15^ While this is a boon to families who can then access these benefits, it is also a major source of geographic disparities in immigrant experiences and health and social outcomes.^16^ Navigating the variable, complex, and dynamic criteria of this patchwork of programs can be overwhelming to newcomers, even more so than for US-born Americans.

Not only are immigrants excluded from many of these programs by strict and confusing eligibility criteria, but the US government has also at times threatened (and acted) to prevent those who participate in these programs from being eligible for permanent residency later. “Public charge” laws allow immigration officials to assess immigrants’ use of public benefits in certain cases as a negative factor in their immigration applications. Most recently, such a public charge law implemented in 2020 was repealed in 2022.^17^ Even so, many immigrants continue to decline to enroll in public benefits because of fears that it would negatively affect their ability to become permanent residents, and even threats of such a public charge law effectively and powerfully discourage vulnerable families from getting the help they desperately need.^18,19^

## Neighborhood placement

Many localities accepting migrants provide shelter to newcomers, ranging from tents to actual housing in a variety of different environments. Numerous studies show that the places where refugees are initially housed matter for their long-term health. For example, previous work has leveraged natural experiments in which refugees to Scandinavian countries in the 1980s–1990s were assigned to neighborhoods across the country in an arbitrary (quasi-random) fashion. At that time, Western and Northern European countries were dealing with an influx of refugees from wars in Eastern Europe, the Middle East, and elsewhere; today's numbers dwarf these prior crises by at least an order of magnitude. In that moment, governments decided to distribute families more evenly across the country to avoid congestion in major cities. Researchers—including our team—tracked these refugees in rich Danish and Swedish registers for decades, linking census data on personal and neighborhood demographic characteristics with individual health data from clinical encounters. We found that children and adults who were assigned to more socioeconomically disadvantaged neighborhoods—with lower area-level income, employment, and education, and more people receiving welfare benefits—had about a 10% increase in the risk of psychiatric diagnoses in the intervening decades.^20^ They were also more likely to have worsened cardiometabolic health, including increased risk of diabetes, hypertension, and myocardial infarction.^21^ These findings add to a larger body of literature on the importance of place-based factors for refugees’ economic outcomes, with one pragmatic study going so far as to develop an algorithmic approach to maximize refugees’ employment prospects in Switzerland by matching them to cities where they were most likely to be well integrated.^22^

The disparities between advantaged and disadvantaged neighborhoods in the United States are even more stark than those in Denmark and Sweden—in part, due to structural racism and disinvestment in racially segregated majority-Black communities, and because of a lack of infrastructure and opportunity in rural areas.^23^ Moreover, Scandinavian countries have more robust and generous support programs than the United States for both low-income groups and the general population (eg, family leave).^24^ Thus, while the results from European studies are not directly generalizable to the US context, they suggest that encouraging the placement of refugees in stable neighborhoods with greater economic opportunity would not only ensure better health in the ensuing years—an important goal in and of itself—but also likely lead to less reliance on medical care and the social safety net. As an aside, while this research was not intended to directly speak to the US context, it also suggests the need for greater investment in racially segregated and economically marginalized US neighborhoods to achieve health equity.

These 3 domains represent evidence-based examples of policies and investments to support the transition of refugees and immigrants to the United States to maximize their own well-being and their ability to contribute to US society. While my personal narrative is the story of a single individual, research shows the imperative of supporting the ability of every immigrant family and child to be healthy and thrive in the Land of Opportunity, for the benefit of us all.

## Supplementary Material

qxae019_Supplementary_Data
